# Estimating Maxillary Sinus Volume Using Smartphone Camera

**DOI:** 10.1109/OJEMB.2024.3516320

**Published:** 2024-12-12

**Authors:** Christoforos Meliadis, Emily Feng, Ezekiel Johnson, Wendy Zhu, Paramesh Gopi, Vivek Mohan, Peter H. Hwang, Jacob Johnson, Bryant Y. Lin

**Affiliations:** University of California 8785 San Francisco CA 94143 USA; San Francisco Otolaryngology Medical Group San Francisco CA 94108 USA; SoundHealth Los Altos CA 94024 USA; Department of Otolaryngology-Head and Neck SurgeryStanford University School of Medicine 10624 Palo Alto CA 94305 USA; San Francisco Otolaryngology Medical Group San Francisco CA 94108 USA; Department of Otolaryngology-Head and Neck SurgeryUniversity of California 8785 San Francisco 94143 USA; Department of MedicineStanford University School of Medicine 10624 Palo Alto CA 94305 USA

**Keywords:** Diagnostic imaging, maxillary sinus, smartphone, telemedicine

## Abstract

*Goal:* This study aims to introduce a novel method for estimating maxillary sinus volume using smartphone technology, providing an accessible alternative to traditional imaging techniques. *Methods:* We recruited 40 participants to conduct a comparative analysis between Computed Tomography (CT) and face scans obtained using an Apple iPhone. Utilizing Apple's ARKit for 3D facial mesh modeling, we estimated sinus dimensions based on established craniofacial landmarks and calculated the volume through a geometric approximation of the maxillary sinus. *Results:* We demonstrated a high degree of agreement between CT and face scans, with Mean Absolute Percentage Errors (MAPE) of 8.006 ± 8.839% (Width), 6.725 ± 4.595% (Height), 9.952 ± 6.733% (Depth), and 10.429 ± 7.409% (Volume). These results suggest the feasibility of this non-invasive approach for clinical use. *Conclusions:* This method aligns with the growing focus on telemedicine, presenting significant reductions in healthcare costs and radiation exposure from CT scans. It marks a substantial advancement in otolaryngology and maxillofacial surgery, showcasing the integration of smartphone technology in medical diagnostics and opening avenues for innovative, patient-friendly, and cost-effective healthcare solutions.

## I. Introduction

The maxillary sinus, a pivotal component in the mid-face region, significantly influences the development of facial contours and dentition. This anatomical feature has been intricately classified into various geometric forms, ranging from semi-ellipsoids to cones [1].

The normal average volume of the maxillary sinus in adults typically ranges from 10 to 20 cm^3^, though individual variations can be considerable. Accurate volumetric assessment of the maxillary sinus is crucial for surgical planning and plays a vital role in several therapeutic and diagnostic applications. For instance, efficient drug delivery into the sinus, especially for conditions like chronic rhinosinusitis, is highly dependent on its volume [2]. Similarly, the efficacy of acoustic resonance therapy for sinus congestion is directly linked to the precise measurement of sinus volume, dictating the therapy's resonant frequency [3]. Moreover, changes in sinus volume are critical markers in grading sinus disease severity and progression, with potential implications in diagnosing obstructive sleep apnea [4].

Computed Tomography (CT) scans have become the gold standard in delineating the intricate anatomy of the maxillary sinus, revealing details about its volume, congestion and the thickness of its bony walls. This modality, along with Magnetic Resonance Imaging (MRI) and Cone Beam Computed Tomography (CBCT), has been pivotal in advancing our understanding of sinus pathologies. Numerous studies have leveraged these techniques to explore the sinus's dimensions and variations, forming the basis for today's diagnostic protocols [5], [6], [7], [8], [9], [10], [11], [12], [13]. However, the limitations of these methods, primarily in terms of accessibility, cost and exposure to ionizing radiation, highlight the need for innovative approaches.

The advent of sophisticated smartphone technology presents an unprecedented opportunity in this realm. Drawing inspiration from craniofacial superimposition techniques [14], our study introduces a novel method utilizing the precision of Apple's ARKit framework for 3D facial mesh modeling to correlate facial landmarks with maxillary sinus dimensions and volume. This approach not only promises to reduce healthcare costs and radiation exposure associated with CT scans but also to revolutionize patient care by providing an accessible and low-cost alternative for maxillary sinus assessment. Future research should focus on integrating functional outcomes, as smartphone technology holds the potential to provide both structural and functional data for comprehensive patient assessment.

## II. Materials and Methods

### A. Participants

We recruited 40 participants, each undergoing a CT scan of their sinuses along with a facial scan using the infrared front-facing camera of an Apple iPhone. This study was conducted under the oversight of the Institutional Review Board with Tracking ID 20201363. Eligible participants were adults over 18 years, fluent in English, and willing to provide informed consent.

We excluded individuals with recent sinonasal or craniomaxillofacial surgeries, active dental infections, acute sinusitis, head-implanted electrostimulation devices, recent head or sinus surgeries, a history of significant intracranial hemorrhage within the last six months, current pregnancy, or any open wounds or rashes on the forehead or scalp.

### B. Computed Tomography

**Fig. 1.**
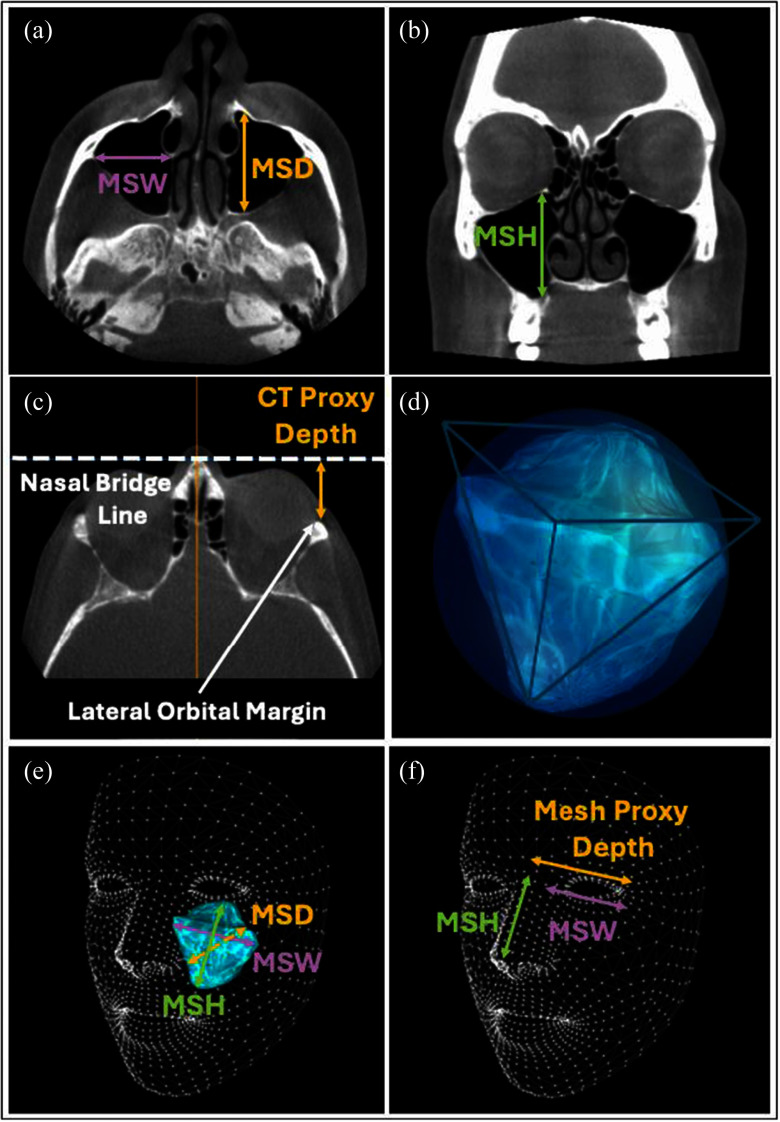
Maxillary sinus measurements. Axial (a) and coronal (b) views of the maxillary sinus were used to calculate its maximal width (MSW), height (MSH) and depth (MSD). Axial view (c) showing the CT proxy depth measured from the lateral orbital margin to the line crossing the nasal bridge. Schematic (d) illustrating the maxillary sinus shape as an intermediate form between a sphere and a pyramid. 3D representation of the left maxillary sinus (e) on Apple's face mesh showing the three actual dimensions of the sinus. Measurements of the maxillary sinus width (MSW), height (MSH) and mesh proxy depth on Apple's face mesh. MSW: Maxillary sinus width, MSH: Maxillary sinus height, MSD: Maxillary sinus depth.

CT scans were conducted using a Xoran MiniCAT scanner, and measurements were performed using OsirixMD software. As described previously [15], the assessment of the maxillary sinus in each patient included bilateral measurements in three planes, performed independently by two reviewers and their average was used for analysis (Fig. 1(a), (b)):

a) Maxillary Sinus Width (MSW) — maximal transverse diameter of the maxillary sinus in the horizontal plane, defined as the longest distance perpendicular from the most prominent point of the medial wall to the most prominent point of the lateral wall as presented on the axial image.
b) Maxillary Sinus Height (MSH) — maximal craniocaudal diameter of the maxillary sinus in the vertical plane, defined as the longest distance from the lowest point of the inferior wall to the highest point of the superior wall as presented on the coronal image.
c) Maxillary Sinus Depth (MSD) — maximal anteroposterior diameter of the maxillary sinus in the sagittal plane, defined as the longest distance from the most anterior point of the anterior wall to the most posterior point of the posterior wall on the axial image.

To ensure accuracy and consistency in these measurements, a protocol for aligning the brain base was employed on the sagittal view, followed by an axial rotation to align the depth landmarks on the coronal view. This additional step in the imaging process was critical for ensuring consistent and balanced assessments of the maxillary sinus dimensions across all scans.

### C. Face Scans

For face scans, Apple's ARKit framework was employed to generate a 1220-dot 3D mesh model of the user's face [https://developer.apple.com/documentation/arkit/arkit_in_ios/content_anchors/tracking_and_visualizing_faces].

This technology allowed for precise estimation of the same maxillary sinus dimensions (MSW, MSH, MSD) as those measured by CT scans. To achieve maximum coherence between the CT scan and the 3D facial scan, we examined and identified facial areas that tend to be the most stable and the least affected by aging and facial fat changes. These break down into 4 facial regions (a) Central orbital region: The central parts of the orbital area remain relatively stable throughout life, even as other areas around the eyes change [16], [17] (b) Bony landmarks: The orbital rims provide more stable reference points (c) Lower facial third: particularly the mandible and chin, tend to change very little in soft tissue compared to other areas. (c) Nasal structure: The nose and its overall bony structure remains relatively stable compared to all other facial features. We then went through a rigorous process of looking at light point and depth coordinate density at each one of these landmarks to choose the final set that were used to train the machine learning algorithm. ARKit points with maximum point density around these landmarks were selected, enabling us to derive the maxillary sinus dimensions from the 3D distances between them (Fig. 1(f)).

After identifying key facial landmarks from the iPhone 3D facial scans, we computed vector distances between selected points. These points were chosen for their high accuracy and consistency across multiple iPhone facial scans. These distances served either as direct measurements or proxies for internal facial landmarks, enabling us to estimate overall volume through a proprietary algorithm.

### D. Depth Estimation Using the Proxy Method

Accurately estimating the depth of the maxillary sinus posed a challenge due to its 3D nature and location deep within the skull, making it not readily measurable from surface scans. To address this, we developed a proxy method for depth estimation by measuring the distance from the lateral orbital margin to the nasal bridge on both CT and face scans (CT Proxy Depth and Mesh Proxy Depth on Fig. 1(c), (f)). This approach was selected for its strong correlation with the actual sinus depth. The method involved finding the ratio between the Actual and Mesh Proxy Depth measurements from the CT, and then applying this ratio to the Mesh Proxy Depth using the following the equation:

\begin{align*}
Estimated\ Depth =& \frac{{CT\ Actual\ Depth}}{{CT\ Proxy\ Depth}}\\
& \times Mesh\ Proxy\ Depth \tag{1}
\end{align*}

This scaling process allowed us to estimate the depth of the maxillary sinus from non-invasive facial scans by leveraging the established relationship between the proxy and actual measurements. Our approach was limited by the standard ARKit set of points that come native to iOS and iPhones with FaceID depth cameras. While we would have ideally liked to get a large depth of field of points that encircled the skull and enveloped the top part of the hairline, we were limited by the ARKit interface and point set. This constraint does indeed limit the accuracy in volumetric calculations.

### E. Volume Estimation

The calculation of each maxillary sinus volume (MSV) was based on the geometric approximation of its shape, which lies between a sphere and a pyramid [15]. Therefore, the average of these two volumes was used for the analysis.

For CT scans, we determined the MSV by averaging the measurements of width (MSW), height (MSH), and depth (MSD) across both the left and right maxillary sinuses. This approach yielded the actual average volume from the geometric approximation of the maxillary sinuses.

For face scans, the estimated volume was calculated using the derived measurements from the 3D mesh model, mirroring the same dimensions and approach.

### F. Statistical Analysis

Our statistical analysis, conducted with GraphPad Prism 10 Software, aimed to evaluate the agreement between CT scans and Face Scans for estimating maxillary sinus dimensions and volume.

Central to our analysis was the Bland-Altman method [18], a robust statistical approach tailored for assessing agreement between two measurement methods. Unlike correlation analysis that assesses the relationship between variables, the Bland-Altman method evaluates how closely two methods agree. In this analysis, we plotted the difference between each pair of measurements against their average. This plot highlights any systematic bias (mean difference) and variability in differences (standard deviation), providing an intuitive understanding of agreement. The 95% limits of agreement, calculated as the mean difference ± 1.96 times the standard deviation, indicate the range within which most differences between the two methods lie. The Bland-Altman analysis is particularly suitable for our study as it quantifies both the average difference and the range of differences between the Face Scan and CT scan measurements. It is a critical tool to ascertain if the face scan can be a reliable substitute for the gold-standard CT scans in clinical settings.

Additionally, we calculated the mean absolute error (MAE) and mean absolute percentage error (MAPE) to provide further perspectives on the measurement accuracy and reliability.

## III. Results

### A. High Degree of Agreement Between CT and Face Scan Measurements

**Fig. 2.**
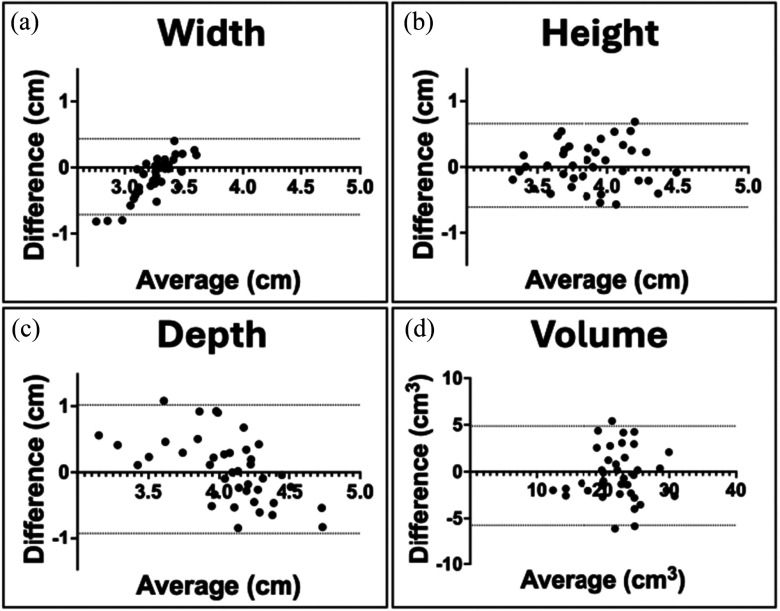
Bland-altman plots for maxillary sinus width (a), height (b), depth (c) and volume (d).

The Bland-Altman analyses for width, height, depth, and volume are visually represented in Fig. 2. These plots underscore the relationship and degree of agreement between the two methods, highlighting a high degree of consistency. The biases and 95% limits of agreement between CT and face scans are presented in Table I.

**TABLE I:** Bland-Altman Analysis

	Bias (Mean ± SD)	95% Limits of Agreement
**Width**	-0.14 ± 0.29	-0.71 to 0.43
**Height**	0.02 ± 0.32	-0.61 to 0.66
**Depth**	0.05 ± 0.50	-0.92 to 1.02
**Volume**	-0.42 ± 2.71	-5.72 to 4.88
		

The mean absolute error (MAE) and mean absolute percentage error (MAPE) for each dimension and volume are presented in Table II. These errors provide additional perspectives on measurement accuracy and reliability.

**TABLE II:** Errors Between CT and Face Scans

	Mean Absolute Error (MAE)	Mean Absolute Percentage Error (MAPE)
**Width**	0.234 ± 0.223 cm^3^	8.006 ± 8.839 %
**Height**	0.263 ± 0.184 cm^3^	6.725 ± 4.595 %
**Depth**	0.406 ± 0.281 cm^3^	9.952 ± 6.733 %
**Volume**	2.222 ± 1.560 cm^3^	10.429 ± 7.409 %
		

These results indicate minimal systemic bias and narrow limits of agreement, suggesting that the face scan method could be a reliable substitute for CT scans in assessing the maxillary sinus anatomy.

## IV. Discussion

Our study successfully demonstrated a significant agreement between maxillary sinus measurements obtained from standard CT scans and those derived using the infrared front-facing camera of an iPhone. This novel application of smartphone technology in medical diagnostics highlights a potential paradigm shift in non-invasive imaging techniques, particularly in the field of otolaryngology and maxillofacial surgery.

The use of smartphone cameras for medical measurements aligns with the growing trend towards telemedicine and accessible healthcare. By leveraging widely available technology, this method offers a practical, cost-effective alternative to conventional imaging techniques, especially in settings where resources are limited, or CT scans are not readily accessible. This approach could significantly reduce healthcare costs and importantly eliminate the radiation exposure risks associated with repeated CT scans.

The advantages of our smartphone-based method include its non-invasiveness, cost-effectiveness, and potential for widespread use given the prevalence of smartphones. This method utilizes Apple's ARKit framework to create a precise 3D facial mesh model, enabling accurate assessment of maxillary sinus dimensions. The high degree of agreement between the CT and face scan measurements validates the reliability and accuracy of this approach.

However, our study also acknowledges certain limitations. The proxy method for depth estimation, while effective, has intrinsic variances that warrant further refinement. The reliance on a single front-camera scan and the need for iOS-compatible technology may restrict the applicability of this method. Future research should explore the feasibility of using multiple scans at various angles to enhance accuracy and expand the technique's use to other smartphone models and operating systems.

Additionally, further studies could investigate the application of this method in various clinical scenarios, including its use in diagnosing and monitoring sinus-related conditions. Research into optimizing the software algorithms and enhancing the accuracy of facial landmark identification could also improve the robustness of this approach. For a more comprehensive clinical approach, future work should aim to incorporate functional outcomes, leveraging the potential of smartphone technology to provide confidence level to the surgeon for future steps and a complete telemedicine solution.

## V. Conclusion

In conclusion, the findings of our research represent a promising advancement in the estimation of maxillary sinus volume, showcasing the potential of integrating smartphone technology into clinical practice. This study serves as a steppingstone towards more innovative uses of mobile technology in healthcare, paving the way for safer, more efficient, and patient-friendly diagnostic methods. It also opens the door for further research into the capabilities of smartphone-based medical imaging, encouraging continued innovation in this rapidly evolving field.
